# Mixed methods evaluation of implementation and outcomes in a community-based cancer prevention intervention

**DOI:** 10.1186/s12889-019-7315-y

**Published:** 2019-08-05

**Authors:** Emily S. King, Carla J. Moore, Hannah K. Wilson, Samantha M. Harden, Marsha Davis, Alison C. Berg

**Affiliations:** 1 Department of Foods and Nutrition, University of Georgia, 202 Hoke Smith Annex, 300 Carlton Street, Athens, GA 30602 USA; 2 Department of Human Nutrition, Foods, and Exercise, Virginia Tech, 1981 Kraft Drive, 1032 ILSB, Blacksburg, VA 24060 USA; 3 Dean’s Office, Department of Health Promotion and Behavior, University of Georgia, 205 Rhodes Hall, Health Sciences Campus, Athens, GA 30602 USA

**Keywords:** Implementation, Consolidated Framework for Implementation Research, Cancer prevention, Extension, Mixed methods

## Abstract

**Background:**

Community-based educational programs can complement clinical strategies to increase cancer screenings and encourage healthier lifestyles to reduce cancer burden. However, implementation quality can influence program outcomes and is rarely formally evaluated in community settings. This mixed-methods study aimed to characterize implementation of a community-based cancer prevention program using the Consolidated Framework for Implementation Research (CFIR), determine if implementation was related to participant outcomes, and identify barriers and facilitators to implementation that could be addressed.

**Methods:**

This study utilized quantitative participant evaluation data (*n* = 115) and quantitative and qualitative data from semi-structured interviews with program instructors (*N* = 13). At the participant level, demographic data (age, sex, insurance status) and behavior change intention were captured. Instructor data included implementation of program components and program attendance to create a 7-point implementation score of fidelity and reach variables. Degree of program implementation (high and low) was operationalized based on these variables (low: 0–4, high: 5–7). Relationships among degree of implementation, participant demographics, and participant outcomes (e.g., intent to be physically active or limit alcohol) were assessed using linear or ordinal logistic mixed effects models as appropriate. Interview data were transcribed and coded deductively for CFIR constructs, and constructs were then rated for magnitude and valence. Patterns between ratings of high and low implementation programs were used to determine constructs that manifested as barriers or facilitators.

**Results:**

Program implementation varied with scores ranging from 4 to 7. High implementation was related to greater improvements in intention to be physically active (*p* <  0.05), achieve a healthy weight (*p* <  0.05), and limit alcohol (*p* <  0.01). Eight constructs distinguished between high and low implementation programs. Design quality and packaging, compatibility, external change agents, access to knowledge and information, and experience were facilitators of implementation and formally appointed internal implementation leaders was a barrier to implementation.

**Conclusions:**

As higher implementation was related to improved participant outcomes, program administrators should emphasize the importance of fidelity in training for program instructors. The CFIR can be used to identify barriers and/or facilitators to implementation in community interventions, but results may be unique from clinical contexts.

**Electronic supplementary material:**

The online version of this article (10.1186/s12889-019-7315-y) contains supplementary material, which is available to authorized users.

## Background

While experts estimate that one third of annual cancer cases could be prevented through lifestyle modifications related to nutrition, physical activity, and weight management [1], approximately 10% of US adults consume the recommended amount of fruits and vegetables [2], 25% meet physical activity guidelines [3], and only 22% are a healthy weight [4]. Furthermore, although the benefits of early detection through cancer screening are well-documented [5–7], many people do not receive recommended screenings [8]. This is particularly true of those without insurance; only 35% of uninsured women regularly get screened for breast cancer and 64% for cervical cancer [8], and colorectal cancer screening use is also lower among men and women without insurance (25%) [8]. Thus, strategies are needed to motivate individuals to change screening, nutrition, and physical activity behaviors to reduce cancer incidence and mortality, specifically among those without insurance.

In response to these needs, the National Breast and Cervical Cancer Early Detection Program (NBCCEDP) was created to provide access to screening for uninsured women 21–64 years of age who are never or rarely screened for breast and cervical cancer [9]. Recognizing that access to services alone does not guarantee utilization, NBCCEDP provides support to state and local health agencies for targeted education and outreach and promotes collaboration with partner organizations, such as the American Cancer Society, to increase program participation [10].

Several interventions [10] have been recognized for their approach to education and positive screening and behavioral outcomes, including the Cooking for a Lifetime of Cancer Prevention (C4L) program [10]. C4L is a community-based educational program with a cooking school format designed to educate women on primary prevention through lifestyle behavior, motivate all women to be screened for breast, cervical, and colorectal cancer, and provide access to breast and cervical cancer screenings for eligible women through the Georgia Breast and Cervical Cancer Control Program (GBCCP, state branch of the NBCCEDP). C4L has reached more than 3500 women over the last 10 years, and has developed a history of practice-based evidence. For example, program evaluation data suggests significant (*P* <  0.05) improvements in intention to implement nutrition and physical activity guidelines for cancer prevention (*unpublished*) and that women who are eligible for BCCP screening services go on to be screened for breast and cervical cancer [10].

While interventions like C4L may initially demonstrate effectiveness for motivating behavior change, a number of other implementation science factors may impact individual- and systems-level changes [11]. Contextual factors, staff turnover, program participant characteristics, and the translation of an intervention from training to actual practice may influence effectiveness of interventions immediately after initial implementation and over time [11, 12]. Furthermore, it cannot be assumed that the intervention will be implemented consistently over time, and that high-quality program delivery will result in the same positive outcomes over time [13, 14]. Accordingly, it is important to periodically evaluate implementation as well as outcomes of interventions that have a long history of practice-based evidence. Implementation science provides an approach through which long-standing interventions may be evaluated, as not all interventions are being translated from efficacy to large-scale dissemination trials.

Taken together, it is not only necessary to understand *if* a program is effective, but it is also necessary to understand the contextual factors that impact delivery [15, 16]. Concurrent evaluation of both implementation and outcomes has multiple benefits including assessment of internal validity, the influence of program drift on participant outcomes, and the translation of interventions to different settings or contexts [11, 14, 15]. Despite these benefits, relatively few studies assess both implementation and outcomes, and challenges exist in comparing implementation across health promotion practice settings [17]. The Consolidated Framework for Implementation Research (CFIR) was designed to overcome some of the challenges and move implementation science forward by synthesizing constructs from several theories into a robust implementation meta-framework [12]. While designed to be used in a variety of contexts and settings, the CFIR has been primarily used to evaluate interventions implemented in clinical care settings, including cancer control programs [18], but has rarely been used to evaluate cancer prevention programs in community settings.

This study was designed to fill a critical gap in the literature by using the CFIR to characterize implementation and to evaluate the relationship between implementation and outcomes in a community-based cancer prevention program with a long history of positive outcomes. The study has the following aims: 1) determine degree of program implementation, 2) determine if implementation is related to outcomes, and 3) use the CFIR to identify barriers and facilitators associated with degree of implementation that may be addressed to improve outcomes.

## Methods

### Study design

This study utilized a mixed methods design that included quantitative participant program evaluations and both quantitative and qualitative semi-structured interviews with the instructors who implemented the program. All methods and procedures were approved by The University of Georgia Institutional Review Board on Human Subjects. Program participants provided written informed consent and instructors provided verbal informed consent.

### Conceptual framework

The CFIR [12] consists of 39 constructs organized into 5 domains that are believed to influence implementation and were collected from various implementation and change theories. For this study, the CFIR was used to develop the instructor interview guide [19] and was the foundation for qualitative data coding and analysis [20]. The analysis methodology was similar to that of Damschroder and Lowery in their evaluation of a weight management program in Veterans Affairs (VA) hospitals [21] and Liang and colleagues’ study of implementing evidence-based cancer control practices in safety net health systems [18].

### Setting

Cooking for a Lifetime of Cancer Prevention (C4L) is a cancer prevention educational program that is disseminated through the framework of the Cooperative Extension System (CES) in Georgia in collaboration with the American Cancer Society (ACS) client navigations in Georgia. The purpose of CES is to translate research and resources from land-grant universities in the United States to communities through educational outreach delivered by community-based Extension professionals [22]. CES is the largest adult educational organization in the US and has, for over 100 years, focused on outreach and service and balanced collection of empirically meaningful data with program evaluation that communicates public value, but does not overburden program participants or Extension professionals, and fits within resource constraints of the Extension system [22].

C4L has been implemented through Georgia Extension for over 10 years in a variety of community-based settings such as churches, technical schools, and non-profit clinics. The program is funded by the American Cancer Society (ACS), administered by state Extension faculty, and implemented by county Extension professionals in collaboration with ACS navigators.

### Program description

C4L is a 2.5-h program that includes three core components: 1) a presentation about breast, cervical, and colorectal cancer screening guidelines given by an ACS client navigator (ACS presentation), 2) a presentation of the ACS guidelines on cancer preventive lifestyle behaviors [23] given by the Extension professional (Extension presentation), and 3) a cooking demonstration with recipe sampling given by the Extension professional (recipe demonstration). ACS lifestyle recommendations discussed include eating a plant-based diet, maintaining a healthy weight, being physically active, and limiting alcohol intake [2]. Extension professionals and ACS navigators work together to recruit for and implement the program. Following the program, ACS navigators assist uninsured female program attendees (21–64 years of age) in applying for breast and cervical cancer screenings through the GBCCP. All participants receive a cookbook as an incentive for program participation. Table 1 is a logic model of the program.

**Table 1:** Cooking for a Lifetime of Cancer Prevention (C4L) Logic Model

Process			Outcomes		
Inputs	Activities	Outputs	Short-term	Intermediate	Long-term
American Cancer Society grant funding USDA GEO00805 NBCCEDP and GBCCP ACS Guidelines on Nutrition and Physical Activity for Cancer Prevention American Cancer Society personnel (director of community outreach, client navigators) Extension personnel (state specialist, administrative assistant, graduate research assistants, county professionals)	Training - Develop training materials - Train client navigators and Extension professionals Implementation - Distribute program materials - Distribute funding - Market program and recruit target audience - Implement 2.5-hr program Evaluation - Collect data from each Extension professional - Analyze program-level and state-level outcomes Preparation of reports	2 organizations involved: American Cancer Society and Extension 6 ACS navigators and 13 Extension professionals trained 24 C4L programs implemented 237 C4L program participants 1 report prepared for funders (ACS) and 9 reports prepared for Extension professionals	Increased awareness of cancer screening recommendations Increased intention to be screened for breast, cervical, and colorectal cancer Increased intention to follow ACS guidelines on nutrition and physical activity for cancer prevention Increased access to breast and cervical cancer screenings for uninsured women	Increased breast, cervical, and colorectal cancer screenings Increased number of individuals who follow ACS guidelines on nutrition and physical activity for cancer prevention Increased number of participants in GBCCP	Decreased breast, cervical, and colorectal cancer incidence and mortality
Contextual Factors (e.g., Affordable Care Act, geographic location, resources, healthcare access, cultural beliefs)					

### Study participants

#### C4L instructors (Extension professionals)

In June of 2016, the state Extension program administrator invited Extension professionals to apply to be C4L instructors via email to an organizational listserv. Extension professionals (C4L instructors) who applied to implement the program during the 2016–2017 grant year and attended program training conducted by Extension state staff were eligible to participate in the study. There were no exclusion criteria for instructors. Thirteen C4L instructors were eligible for study participation and were recruited during a scheduled one-on-one telephone call to the research team prior to program implementation. All 13 eligible instructors agreed to participate, were scheduled for an interview, and received a $30 credit for work supplies upon completion of the interview. Verbal consent was obtained before the interview began.

##### Instructor training on program implementation

C4L instructor training was 1 day (8 am to 3 pm), and included Extension professionals (C4L instructors), ACS navigators, and program administrators (Extension and ACS). Training addressed implementation of core components of the program, the importance of program fidelity, and other critical aspects of implementation. The three core program components include: ACS presentation, Extension presentation, and recipe demonstration. Other essential components of implementation include: recruiting the target audience (women without insurance aged 21–64), providing and explaining research study consent forms, administering the program evaluation, and offering participant incentives (cookbooks and two, $25 gift cards or prizes of equal value). C4L Instructors were the lead implementers of the program and responsible for ensuring all components were implemented as described, including recruitment of the target audience. ACS navigators were expected to help with recruitment of the target audience and implementation of the ACS presentation, and may help with other parts of implementation as agreed upon between the C4L instructor and the ACS navigator. The training agenda is available as Additional file 1.

#### C4L program participants

C4L instructors, ACS navigators, and/or staff at the location where the program was delivered (i.e. church staff, non-profit clinic staff) recruited C4L program participants through print media, social media advertisements, email listservs, and word of mouth. The goal of recruitment was to reach the target audience, women eligible for GBCCP screening services, (uninsured, ages 21 to 64 y), but women and men of all ages were invited to attend the program. Inclusion criteria for the present analyses were: participation in one of the 13 programs evaluated and age 21 and older. There were 139 participants in the 13 programs included in the present study. Eleven participants were missing information on age and sex and three were less than 21 years of age and thus, were excluded from the sample. Because the program focuses primarily on female cancer screening and male participation was low (*n* = 10), males were also excluded from the sample. Thus, the final analytical sample for this study (*n* = 115) included only women aged 21 and older who attended the 13 programs evaluated in the interviews. All program participants included in the analysis provided written informed consent for research.

### Data collection

#### Interviews with C4L instructors

One-on-one, semi-structured interviews were conducted with C4L instructors using Zoom Web Conferencing (Zoom Video Communications, Inc., San Jose, California) within 0–4 weeks of program implementation (mean 9.5 [6.7] days from implementation, range 0–22 days) and lasted 61.5 [16.1] minutes. Instructors implemented from one to five programs in the 2016–2017 grant year, with the majority (62%) implementing only one program. However, to make cases comparable, instructors were only interviewed about the first program completed. Participants were told they could refuse to answer any question or stop the interview at any time. Audio files of the interviews were transcribed by a third-party transcriptionist (Rev.com, San Francisco, California). A research team member reviewed transcripts for clarity and accuracy and coded identifying information.

The interview guide included two types of questions. Closed-ended interview questions (quantitative data) assessed completion of critical program components (e.g., Was the Extension presentation given? Were recipes demonstrated?). Open-ended interview questions (qualitative data) were developed using the CFIR website resources [19] and explored perceptions of training and implementation. The interview guide is available as Additional file 2.

#### C4L participant program evaluation

C4L program participants completed a retrospective, researcher-designed questionnaire at the conclusion of the program while recipes were sampled and before incentives were provided. The questionnaire included demographic and health insurance information and intention to engage in cancer preventive screening, nutrition, and physical activity behaviors before and after the program, and is discussed further in the next sections.

### Measures

#### Program implementation measures

##### Program reach

C4L instructors submitted a report of program attendance. Reports and participant evaluations (sex, age, health insurance information) were used to determine total attendance for each program (total participants attended) and target audience in attendance (uninsured, women 21–64 years). Target audience reach (%) was calculated for each program (reach = [target audience in attendance / total attendance] × 100%).

##### Degree of implementation

Degree of implementation (high v. low) was determined using a 7-point scale of reach, fidelity, and critical implementation components. Each component was worth one point: target audience, ACS presentation, Extension presentation, consent forms, evaluations, recipe demonstration, and program incentives. Target audience reach was calculated as described above, and the benchmark for implementation was 50% or greater to indicate that a program included a majority of participants from the intended target audience (uninsured women ages 21 to 64 y). Completion of the other components was assessed via closed-ended interview questions with C4L Instructors and interview context as appropriate. For example, if an instructor answered that a component was completed (e.g., recipe demonstration), but it was revealed later in the interview that it was not completed in its entirety (e.g., recipes were sampled, but there was no cooking demonstration), a point was not given. Similar to other researchers [18], we categorized the programs into low or high implementation programs. Low implementation was determined to be a score of 5 or lower (missing two or more of the critical program components) according to the spread of the data (Median score: 5, range 4–7), and to indicate a program that was not implemented as intended which we hypothesize may impact outcomes.

#### Program outcome measures

##### Nutrition and physical activity behaviors

Participants indicated how likely they were to implement cancer preventive nutrition and physical activity behaviors *before* and *after* the program using a 5-point Likert-type scale (1 = not at all likely, 5 = extremely likely). Seven cancer preventive behaviors discussed in the program were assessed: be physically active for at least 30 min 5 days or more a week, achieve a healthy weight, limit alcohol (≤1 drink/day for women or ≤ 2 drinks/day for men), fill half your plate with fruits and vegetables, choose whole grains at least half of the time, eat ≤18 oz of red meat per week, and avoid processed meat. Change variables were created by subtracting the *before* response from the *after* response.

##### Cancer screening behaviors

History of cervical, breast, and colorectal cancer screening was measured by participants answering when their last Pap test, mammogram, Fecal Occult Blood Test (FOBT)/Fecal Immunochemical Test (FIT), and colonoscopy were, respectively. Answer choices were informed by US Preventive Services Task Force recommendations [6, 7, 24] to identify individuals never and rarely screened and were “never,” “within the past 3 or 10 years” (3 years for Pap test, mammogram, and FOBT/FIT, and 10 years for colonoscopy), “more than 3 or 10 years ago,” and “do not know.” Intention to get screenings after the program was measured by a 3-point scale, from “Not at all” to “I’m definitely going to do it.”

### Statistical analysis

Relationships among degree of implementation (high/low), demographic variables, and change variables for nutrition and physical activity behaviors were explored using linear mixed effects models to incorporate potential variability at the program level (implementation) along with the participant-level variables (demographics and behavior change intention). Event number was used as a random effect in the models. Independent variables included in each model were implementation level, age, race, ethnicity, education, and insurance. Type III F tests were conducted, and denominator degrees of freedom were determined using the Satterthwaite method for mixed effects models. Post-hoc analyses were conducted for significant independent variables in the models, which included calculation of estimated marginal means or beta coefficients and pairwise comparisons using Least Significant Differences where appropriate. All model residuals were tested for normality through histograms and visualization.

Ordinal logistic regression mixed effects models were used to explore relationships among implementation level, demographics, and intention to be screened for cancer (cervical, breast, and colorectal). Only participants who were in the appropriate age range for each test were included in the statistical analysis (Pap test: aged 21 to 64 [*n* = 59], mammogram: aged 40 and older [*n* = 76], FOBT/FIT and colonoscopy: aged 50 and older [*n* = 49 and 59, respectively]). Independent variables included in each model were implementation level, age, race, ethnicity, education, insurance, implementation of ACS presentation, and participant screening history. Models were adjusted as necessary to accommodate low variability in responses.

All analyses were conducted using IBM SPSS Statistics version 25 (Armonk, New York). Results are presented as means and standard errors (M [SE]), and 95% confidence intervals (95% CI) as appropriate.

### Interview analysis

#### Data coding

The methods for interview data coding and analysis were adapted from Damschroder and colleagues [12, 21, 25, 26]. Transcript coding was largely deductive based on CFIR constructs [12], but inductive coding was used when it was determined that a CFIR code did not adequately explain the aspect of implementation described. All CFIR domains were considered during coding. Analyst triangulation occurred through the use of a consensual qualitative research approach – three members of the research team coded all transcripts independently then met to reach consensus on any differences [27]. The first three transcripts were coded by the entire team to operationalize construct definitions. Subsequent transcripts were independently coded by balanced pairs of analysts to reduce the impact of individual bias. Between each round of coding, the codebook was updated by the lead analyst and approved by the team. After coding was completed, two analysts completed an audit of the final codes together to ensure consistent and accurate use of the constructs. ATLAS.ti version 8 was used as a tool for coding and analysis (Scientific Software Development GmbH, Berlin, Germany).

#### Construct ratings

Following coding, the research team rated the constructs on a scale of − 2 to + 2 to indicate influence on implementation [21, 25, 26]. The sign indicated valence (positive or negative influence on implementation) and number indicated magnitude (strength within an interview). Zero indicated no influence on implementation (neutral), and an “X” indicated both positive and negative influences (mixed) [19, 25, 26]. The average frequency of construct use across all interviews was used to approximate magnitude, i.e., above average frequency of the construct was rated as 2 instead of 1. A variable-oriented approach (rating one construct at a time) was used to maintain consistent application of ratings across a construct [21]. Rating was done individually by the analysts before meeting as a group to reach consensus.

#### Analysis and interpretation

The final ratings for each construct present in each interview were summarized in a ratings matrix by the lead analyst, with programs grouped according to degree of implementation (high or low). Using this matrix, the research team visually identified patterns in the construct ratings that distinguished between high and low implementation programs [18, 21]. Based on this assessment, constructs were determined to be not distinguishing between programs with high or low implementation (no discernible pattern), or distinguishing, which was further classified to weakly distinguishing (pattern observed but mixed positive and negative valence ratings present, or only slight difference between magnitudes) or strongly distinguishing (clear difference in valence and/or magnitude) through a consensual qualitative approach. Distinguishing constructs were then determined to be barriers to or facilitators of implementation or descriptive only using valence and interview text.

## Results

### Study participant characteristics

#### C4L program participants

Participants were female, primarily middle-aged, African American, and non-Hispanic (Table 2). Most participants (88.5%) had at least a high school education and some type of health insurance (82%).

**Table 2:** C4L program participant characteristics

Demographics	*n*	Mean (SD) or %
Age	115	56.6 (14.4)
Race	111	
White		28.8
Black		67.6
Asian		0.9
Other		2.7
Ethnicity	102	
Not Hispanic		90.2
Hispanic		9.8
Education	104	
Grades 1–8		1.0
Grades 9–11		10.6
Grade 12 or GED		29.8
College 1–3 years		35.5
College 4 or more years		23.1
Health Insurance	109	
Has health insurance		75.2
No health insurance		24.8

#### Interview participants

C4L instructors had a mean 13.4 (8.7) years of experience in Extension, 11.0 (7.6) years in FACS Extension, 9.2 (9.3) years in their current county, and 2.4 (3.1) years of experience with the C4L program.

### C4L program outcomes and implementation

An average of 8.8 (6.1) participants attended each program (range 4 to 25), and average target audience reach was 21.1% (22.1) (range 0.0 to 66.7%).

Of the 13 programs, 6 had high implementation and 7 had low implementation (Table 3). Of the 7 implementation components, meeting the target audience was the most commonly missed followed by completion of the ACS presentation.

**Table 3:** C4L program implementation scores (*N* = 13)

Implementation Components^a^									
Program	Target Audience > 50%^b^	ACS Presentation	Extension Presentation	Consent Forms	Program Evaluations	Recipe Demonstration	Incentives	Total Score	High / Low
A			X	X	X		X	4	Low
B		X	X	X	X	X		5	Low
C			X	X	X	X	X	5	Low
D			X	X	X	X	X	5	Low
E		X	X	X	X		X	5	Low
F			X	X	X	X	X	5	Low
G			X	X	X	X	X	5	Low
H		X	X	X	X	X	X	6	High
I		X	X	X	X	X	X	6	High
J		X	X	X	X	X	X	6	High
K	X	X	X	X	X	X	X	7	High
L	X	X	X	X	X	X	X	7	High
M	X	X	X	X	X	X	X	7	High

^a^ “X” indicates program components that were completed

^b^ More than 50% of the total program participants met intended target audience criteria: uninsured women, 21–64 years

### Relationship between C4L implementation, participant characteristics, and participant outcomes

#### Participant intention to implement cancer preventive nutrition and physical activity behaviors

Table 4 presents the general linear mixed effects models for the change in intention to implement cancer preventive nutrition and physical activity behaviors, and Table 5 presents post-hoc testing results. Implementation was significantly related to change in intention to be physically active (*p* = 0.016), achieve a healthy weight (*p* = 0.034), and limit alcohol (*p* = 0.009). Participants in both high and low implementation programs significantly increased their intention to be physically active and achieve a healthy weight as a result of the program, but those in high implementation programs reported a significantly greater change (2.63 [0.55] and 2.12 [0.52], respectively) than those in low implementation programs (1.28 [0.45] and 1.39 [0.42], respectively). While participants in high implementation programs significantly increased intention to limit alcohol (0.82 [0.38]), those in low implementation programs did not experience a significant change (0.16 [0.31]). Implementation was not significantly associated with change in intention to change other behaviors.

**Table 4:** General linear mixed effects models for changes in nutrition and physical activity intentions

Behavior Independent variables	Numerator df	Denominator df	F statistic	*p* value <
Be physically active for at least 30 min 5 days or more a week (*n* = 78)				
Implementation	1	7.08	9.85	0.05
Age	1	65.42	0.01	NS
Race	2	66.22	0.13	NS
Ethnicity	1	36.92	3.71	NS
Education	3	63.37	2.68	NS
Health insurance	1	68.00	4.57	0.05
Achieve a healthy weight (*n* = 75)				
Implementation	1	65	4.72	0.05
Age	1	65	0.31	NS
Race	2	65	0.19	NS
Ethnicity	1	65	3.25	NS
Education	3	65	0.47	NS
Health insurance	1	65	4.04	0.05
Limit alcohol: 1 drink or less per day for women and 2 or less for men (*n* = 78)				
Implementation	1	68	7.13	0.01
Age	1	68	1.55	NS
Race	2	68	1.25	NS
Ethnicity	1	68	0.16	NS
Education	3	68	2.28	NS
Health insurance	1	68	4.63	0.05
Fill half your plate with fruits and vegetables (*n* = 78)				
Implementation	1	68	2.74	NS
Age	1	68	1.35	NS
Race	2	68	0.85	NS
Ethnicity	1	68	2.29	NS
Education	3	68	0.41	NS
Health insurance	1	68	1.50	NS
Choose whole grains at least half of the time (*n* = 76)				
Implementation	1	66	3.75	NS
Age	1	66	0.92	NS
Race	2	66	0.31	NS
Ethnicity	1	66	0.02	NS
Education	3	66	0.72	NS
Health insurance	1	66	0.60	NS
Eat 18 oz or less of red meat per week (*n* = 75)				
Implementation	1	65	0.41	NS
Age	1	65	5.94	0.05
Race	2	65	1.03	NS
Ethnicity	1	65	0.37	NS
Education	3	65	4.24	NS
Health insurance	1	65	5.94	0.05
Avoid processed meat (*n* = 74)				
Implementation	1	7.06	3.94	NS
Age	1	63.78	4.67	0.05
Race	2	63.37	0.47	NS
Ethnicity	1	34.46	0.47	NS
Education	3	60.57	3.00	0.05
Health insurance	1	63.61	18.51	0.001

*NS p* value not statistically significant (> 0.05)

**Table 5:** Post hoc analyses for significant effects of implementation and insurance on behavior change intention

Behavior Independent variables	Mean	Std. Error	df	95% Confidence Interval	*p* value
Be physically active for at least 30 min 5 days or more a week (*n =* 78)					
Implementation					
Low	1.28	0.45	21.7	0.34, 2.22	0.010
High	2.63	0.56	19.8	1.50, 3.77	< 0.001
Has insurance					
No	1.61	0.48	30.54	0.64, 2.58	0.002
Yes	2.30	0.48	33.35	1.32, 3.29	< 0.001
Achieve a healthy weight (*n* = 75)					
Implementation					
Low	1.39	0.42	65	0.55, 2.24	0.002
High	2.12	0.52	65	1.10, 3.15	< 0.001
Has insurance					
No	1.39	0.46	65	0.47, 2.32	0.004
Yes	2.13	0.48	65	1.15, 3.10	< 0.001
Limit alcohol: 1 drink or less per day for women and 2 or less for men (*n* = 78)					
Implementation					
Low	0.16	0.31	68	−0.45, 0.78	0.602
High	0.82	0.38	68	0.07, 1.57	0.032
Has insurance					
No	0.21	0.34	68	−0.47, 0.88	0.546
Yes	0.78	0.36	68	0.07, 1.49	0.032
Eat 18 oz or less of red meat per week (*n* = 75)					
Has insurance					
No	0.42	0.48	65	−0.55, 1.39	0.390
Yes	1.20	0.51	65	0.18, 2.22	0.022
Avoid processed meat (*n* = 74)					
Has insurance					
No	−0.50	0.54	29.51	−1.60, 0.60	0.358
Yes	1.16	0.55	33.65	0.034, 2.29	0.044

Insurance was also associated with change in intention to implement several nutrition and physical activity behaviors (Table 4) such that those with insurance were more likely to increase intention as a result of the program (Table 5).

#### Participant intention to get cancer screenings

Table 6 presents participant intention to get cancer screenings after attending C4L. Table 7 presents regression models of the relationship between implementation and intention to be screened for cancer. Overall, participants intended to get cancer screenings, but degree of implementation was not significantly associated with intention to be screened. However, implementation of the ACS presentation alone was significantly associated with intention to get an FOBT/FIT (*p* = 0.029). Those who saw the complete ACS presentation were significantly less likely to intend to get an FOBT/FIT than those who did not (OR 0.1155, 95% CI 0.02 to 0.79).

**Table 6:** Intention to get cancer screenings after attending C4L

	*n*	Not at all (%)	I may do it (%)	I’m definitely going to do it (%)
Pap test	59	3.4	11.9	84.7
Mammogram	76	5.3	6.6	88.2
FOBT/FIT	49	12.2	42.9	44.9
Colonoscopy	59	6.8	20.3	72.9

**Table 7:** Ordinal logistic regression mixed effects models for changes in screening behavior intentions

Screening test Independent variables	Numerator df	Denominator df	F statistic	*p* value <
Pap test (*n =* 59)				
Implementation	1	42	0.05	NS
Age	1	42	0.26	NS
Race	1	42	0.34	NS
Education	3	42	0.52	NS
Health insurance	1	42	0.41	NS
ACS presentation implementation	1	42	0.94	NS
Screening history	1	42	1.34	NS
Mammogram (*n =* 76)				
Implementation	1	61	0.14	NS
Age	1	61	0.45	NS
Race	1	61	2.18	NS
Education	3	61	0.72	NS
Health insurance	1	61	0.34	NS
ACS presentation implementation	1	61	0.27	NS
Screening history	2	61	0.59	NS
FOBT/FIT (*n* = 49)				
Implementation	1	35	0.00	NS
Age	1	35	3.20	NS
Race	2	35	0.45	NS
Education	3	35	0.44	NS
Health insurance	1	35	2.37	NS
ACS presentation implementation	1	35	5.17	0.05
Screening history	3	35	2.87	NS
Colonoscopy (*n* = 59)				
Implementation	1	7	0.10	NS
Age	1	44	0.74	NS
Race	2	44	2.51	NS
Education	3	44	1.18	NS
Health insurance	1	44	0.05	NS
ACS presentation implementation	1	6	0.14	NS
Screening history	2	44	2.41	NS

*NS p*-value not statistically significant (> 0.05)

### Distinguishing implementation constructs from interviews: barriers and facilitators

Qualitative data aligned with 29 CFIR constructs [12] and four additional constructs were created by the analysts during coding: agent networks, C4L experience, extension promotion, and time. For the present analysis, the CFIR construct formally appointed internal implementation leader [12] was renamed formally appointed *external* implementation leader to describe the role of the ACS navigators. As described by Damschroder et al., (2009), formally appointed internal implementation leaders are individuals within the implementing organization assigned to implement the program but it is not explicitly part of their job. In the context of this study, the ACS navigators are appointed by ACS to help recruit for the programs and present the screening guidelines, but it is not a part of their formal job description and they are *external* to Extension (the inner setting in this analysis).

The distinguishing constructs and their ratings across programs are presented in Table 8. Eight constructs were found to be distinguishing between high and low implementation programs, with three strongly distinguishing. The other constructs were either not present in enough interviews or had mixed influence on implementation. Representative quotes from both high and low implementation programs for all distinguishing constructs are presented in Table 9. The next sections discuss distinguishing constructs, organized by CFIR domain, and whether they presented as a barrier or facilitator to implementation.

**Table 8:** Ratings matrix of distinguishing constructs from qualitative analysis

	Low Implementation Programs							High Implementation Programs						
	A	B	C	D	E	F	G	H	I	J	K	L	M	
Intervention Characteristics														
Design Quality & Packaging	+ 1	+ 1	+ 1	+ 1	X	+ 1	+ 2	X	−1	+ 1	+ 1*	+ 2	X	*
Inner Setting														
Compatibility	+ 2	+ 1	+ 2	+ 1	+ 2	+ 1	+ 1	+ 1	+ 1	+ 1	+ 1	+ 1	+ 2	*
Access to Knowledge & Information	+ 2	0	+ 1	+ 1	X	−1	+ 1	+ 1	+ 1	+ 1	+ 1	− 1	+ 1*	*
Characteristics of Individuals														
Other Personal Attributes	+ 1	M	+ 1	M	+ 1	+ 2	0	−1	+ 1	M	+ 2	− 1	X	*
Process														
Executing	X	−1	+ 1*	−1	−2	− 1	−1	− 1	X	+ 1	+ 1	+ 1	− 1	**
External Change Agents	+ 1	+ 1	+ 2	M	0	X	+ 1	+ 1*	+ 2	+ 1	+ 2	M	+ 2	**
Formally Appointed External Implementation Leader	+ 1	−1	−1	−1*	M	−2	−2	−1	− 1	−1	X	−1	+ 1*	*
Other														
Experience	−1	+ 1	+ 1	0	M	−2	M	+ 1	M	+ 2	+ 2	−2	+ 1	**

Key:Rating + or – indicates valence of the construct on implementation, and 1 or 2 indicates the magnitude of the construct in the program; * after the rating indicates that the construct was mixed but was slightly more positive or negative; 0 represents a neutral influence on implementation; X indicates mixed influence on implementation; M indicates missing; construct was not present in the interview; * in the final column indicates a weakly distinguishing construct; ** in the final column indicates a strongly distinguishing construct

**Table 9:** Representative quotes from low and high implementation programs

Construct (CFIR Domain)	Low Implementation Programs	High Implementation Programs
Design Quality & Packaging ^a^ (Intervention Characteristics)	“… I had the knowledge and definitely the materials, the website that you guys provide with all of the resources is probably, out of all the programs that we offer, probably the most concise one that we have”. … “is the most concise, the most organized program that we have. Like I don’t like when I have to go teach a program and like have to create like an extra piece on my own.” – Participant G	“Um … If the evaluation could go down to two pages. I know that’s really difficult. … I think some of the recipes in the cookbook are a little, they seem a little complicated and like a lot of re-, a lot of ingredients. And sometimes I feel like they’re not necessarily the healthiest options.” – Participant H “I think it was one of the few things that I felt was packaged enough and ready to go, that this was something I could go out into the community and offer without a lot of synthesis. Because I feel like a lot of times I am making up presentations or whatever, and this had such a clear goal that had data attached to it, that I think was a good thing to do, especially early on.” – Participant L
Compatibility ^a^ (Inner Setting)	“Cancer Cooking School fits very well into programming for most agents who are involved in chronic disease prevention, so it’s just a very smooth and easy transition where can get a little extra funding and we don’t have to force it into the mold.” – Participant E	“Oh, well I think it fits perfectly within the scope of work and the goals of FACS programming. One of our main focuses is chronic disease prevention so it fits right in with that and in terms of the nutrition education, it goes with it. A lot of that is information that we already teach. Some of it’s expanded on, which is a good thing. It allows us to get a little bit more depth on some things, so I think it fits perfectly.” – Participant M
Access to Knowledge & Information ^a^ (Inner Setting)	“I was looking at the website today because I went to print out the coversheet. And the only thing it says, that I noticed about planning ahead, was 2 to 5 days call the program leader. And make sure you understand the evaluation process … Looking back now, the evaluation and consent forms, that was the easiest part of the entire thing. … There’s a lot of moving parts to this thing.” – Participant F	“And the training that was in November, in Macon, um, was really good because the program leader had updated some stuff, so it was good to see that. Um, and it was really good to see you, actually, do the school. That was helpful.” – Participant H
Other Personal Attributes ^a^ (Individual Characteristics)	“I think there’s two main factors. I think the training was a large part of it. It was the training and then combined with my experience in Extension and teaching and teaching programs around nutrition and health that I felt pretty good about this subject matter already.” – Participant A	“I was really excited waiting for 30 people, um, I was happy to have a lot of people for the program but it didn’t happen.” – Participant K
Executing ^b^ (Process)	“You know the only thing is just being kind of loose with the target audience. That was a the biggest deal. Like I said, we had one that I’m pretty sure, maybe two, that fit that target audience and the rest you know they just didn’t.” – Participant E “Honestly, we didn’t spend a lot of time in the handouts.” – Participant E	“I think registration went really smoothly … And I think the recipe selection was great and people enjoyed it. And they got the educational part of the recipe but then they also liked the taste and the flavor. Um, and there was a good size group. It wasn’t too small and it wasn’t too large, so there was good communication and discussion throughout the actual event. And, overall, everyone seemed to enjoy it.” – Participant H
External Change Agents ^b^ (Process)	“Once I had the flyer, I used the flyer that you all had created, but I kind of Frankenstein-ed it into one that really worked for me. They [the external change agent] did not want to promote it as a cancer prevention cooking school. They thought cancer in the title would put people off.” – Participant F	“I chose five because I did a-I did a committee effort. So I had other organizations join in to help with the marketing, they also help with registration. They help with planning, securing the location, all the logistics. … Yes, it worked well for the partners who are heavily instrumental to and getting this program out to the community.” – Participant I
Formally Appointed External Implementation Leaders ^a^ (Process)	“So, she preferred to use her own PowerPoints, the American Cancer Society PowerPoint but she used her own because they had certain photos in them and she preferred to show the photos that were more detailed about breast cancer, for instance … Her PowerPoint slides were more detailed and longer, so she went through the breast cancer, she went through the colon cancer screening very well. She did not do a portion of the presentation about the Pap test.” – Participant A	“She was very engaged and very responsive and we communicated a good bit early on to select a date and get her familiar with the group and that kind of thing, and then again right before the school just to confirm the details.” – Participant M
Experience ^b^ (Other)	“But I mean I’m not, it’s not a bad thing, it’s just one of those things, until you actually go through the process you’re really not gonna know how long it takes.” – Participant F	“I think when you have it all laid out the way you’re supposed to teach it, and I guess, too, I’ve taught it so many other times, it’s not like it’s something totally new. It may be we change some material very year to keep up with the changes with the statistics and things like that. But other than that, it’s not a big thing for me.” – Participant J

Key:^a^ Weakly distinguishing^b^ Strongly distinguishing

#### Intervention characteristics

##### Design quality & packaging

Design quality and packaging was a weakly distinguishing construct that facilitated implementation overall. Interview participants thought that the program materials were effective and well-organized. Some instructors, particularly from the high implementation programs, pointed out that the program evaluations were long and participants had difficulty understanding the questions. Another topic was the need to update the cookbook, the incentive for attendees.

#### Inner setting

##### Compatibility

This CFIR construct was weakly distinguishing and generally expressed as a facilitator of implementation. All of the C4L instructors interviewed work in nutrition and chronic disease prevention; therefore, this program is very compatible with their work. While all interview participants agreed that the C4L program is compatible, interviewees from the low implementation programs emphasized compatibility more.

##### Access to knowledge & information

Access to knowledge and information refers to the “ease of access to information about the innovation,” which was training and online instructional materials in our analysis [16]. Access to knowledge was weakly distinguishing. The instructors from high implementation programs noted the program training and the website instructional materials helped them feel prepared and facilitate program implementation. The low implementation program instructors expressed that additional information would have been helpful (i.e., knowing how much time it takes to prepare for the program). Thus, for low implementation programs, this construct was a barrier to implementation. For example, Participant A said:

*… You might in the training, pair them up with an experienced [C4L instructor] and let them- let the experienced [ instructor] kind of go through the logistics and talk to them about um, what it might require of them. … how much time it might take and- and all of that stuff.*

#### Characteristics of individuals

##### Other personal attributes

Other personal attributes was a broad construct that manifested in multiple ways throughout the interviews. The construct was weakly distinguishing, with more positive ratings in the low than high implementation programs. However, it was used to describe characteristics such as tolerance for stress, organizational skills, and general work experience. Thus, depending on the attribute, it served as either a barrier or facilitator.

#### Process

##### Executing

Executing was found to be strongly distinguishing. Executing described accurate implementation of the program according to the training; it was not a facilitator or barrier to implementation but instead described implementation. Some examples included using the screening tool to recruit individuals within the target audience, keeping the program on time, completing a recipe demonstration, and providing the correct incentives. The low implementation programs had more negative ratings overall.

##### External change agents

External change agent construct was strongly distinguishing and a clear facilitator of implementation. External change agents were community partners that helped to plan, recruit, and/or host the program. Working together with these organizations facilitated implementation, particularly reaching the target audience. Only one of the high implementation programs did not have a community partner. Among the low implementation programs, some did not have a community partner and others had partners that were not engaged throughout program planning or recruitment.

##### Formally appointed external implementation leaders

Formally appointed external implementation leaders was a weakly distinguishing construct. The ratings for this construct were mostly negative, but programs with low implementation had more negative ratings. For example, Participant F said,

*As far as the planning went, ACS navigator 205 didn’t really have anything to do with the planning after the initial meeting … I don’t think she brought enough materials. And she knew how many people were gonna be there.*

C4L instructors expressed a desire to improve the collaboration with the ACS navigators, as overall, they were discussed as barriers to implementation.

#### Other emergent constructs

##### Experience

Experience was found to be a facilitator of implementation and strongly distinguishing between the high and low implementation groups. Among the programs with high implementation, only one C4L instructor did not have prior experience with the program. In contrast, four instructors in the low implementation group had never implemented C4L. Those with prior program experience noted that implementing the program previously gave them greater confidence for implementation. Those who had not completed the C4L noted that they did not anticipate how much time was needed to prepare.

## Discussion

This study provides new information about relationships between implementation and program participant outcomes as well as rich descriptions of CFIR constructs that manifested as barriers and facilitators to implementation of a community-based cancer prevention program with a long history of generally positive participant outcomes. Despite all instructors receiving the same program training, implementation materials, and access to implementation instructions on the web and program administrators for support, our study found variation in program implementation.

Notably, a higher degree of fidelity in implementation was related to participants’ intentions to be physically active, achieve a healthy weight, and limit alcohol. It is uncertain why an effect was not found for the other behaviors discussed in the program (i.e., avoiding processed meat, filling half of plate with fruits and vegetables). As desired outcomes may be achieved by reaching a threshold of implementation or improve as implementation improves [14, 28], it is possible that some aspect of implementation that was relevant to these outcomes was not captured in the implementation measures. In contrast, implementation of the cancer screening presentation was inversely related to intention to get an FOBT/FIT. The cancer screening presentation provides information about FOBT/FIT, a stool sample test done at home, that may deter individuals. Low variability in response for intention to get the other cancer screenings likely limited statistical power to detect any relationship between implementation and intention to get a Pap test, mammogram, or colonoscopy.

Although the primary focus of the analysis was implementation, it is important to note that results suggest the program is more effective for individuals with insurance. Participants with health insurance had significantly greater changes in intention to implement several lifestyle behaviors than those without insurance. Appropriateness and acceptability are key implementation outcomes, so evaluating participant characteristics and outcomes can ensure that an intervention is effective for its intended audience [17]. These results suggest that the C4L program may need revisions to better reach its target audience: women without insurance.

The C4L instructor interviews added depth to the study by describing what specific barriers and facilitators contributed to the varied degree of implementation. CFIR constructs that manifested as barriers and facilitators to implementation were similar to other studies, but some nuanced differences are of note. Like other studies, access to knowledge and information, compatibility, and design quality and packaging were found to be facilitators of implementation [21, 26, 29, 30]. However, these constructs distinguished between high and low implementation programs in our study where they were not distinguishing in others [21, 25, 26, 29, 30]. One possible explanation for this difference is related to another distinguishing construct, experience. The C4L program has been in place for over a decade, whereas other interventions evaluated using CFIR had been implemented for only a few months to a few years [18, 21, 26, 29, 31]. Therefore, the instructors in our study had varying levels of program experience; and in general, those with more experience had high implementation and a different perception of implementation than those with less experience. For example, while all instructors spoke of compatibility as a facilitator of implementation, those with low implementation (less experience) had more positive comments about compatibility than those with high implementation, which made the construct distinguishing. It is possible that new instructors emphasized compatibility more because they were justifying their reasons for being involved. More senior instructors mentioned compatibility positively, yet very briefly, as a reason for sustained implementation. The low implementation group also had more positive comments about design quality and packaging compared to those with high implementation, who viewed design quality and packaging as a facilitator to implementation but provided more constructive feedback. Having more experience may have contributed to a deeper understanding of program materials and how they could be improved. This suggests that administrators of longstanding programs should consider the effect of the interventionist’s experience when assessing implementation or designing an implementation study. Moreover, using CFIR as the framework to evaluate more established programs may yield different results than when used to evaluate newer interventions.

Another key finding that differed from previous studies [18, 21, 26, 29] was the impact of collaboration with other organizations on implementation. While in previous literature external change agent was completely absent from analysis [18, 21, 26] or had a mixed influence on implementation [29], external change agents was a strongly distinguishing construct and facilitated implementation, specifically recruitment, in this study. This study also had formally appointed *external* implementation leaders (ACS navigators) who were described as barriers to implementation. While the effect of formally appointed internal implementation leaders on implementation has been mixed [21, 26, 29], it was not surprising that the external implementation leader construct was distinguishing in this study due to their roles in recruitment and implementation. In a clinical setting where an intervention is contained within a department or hospital unit and program participants may be referred by a health care provider, collaboration with outside organizations may not be as influential. In a community context, however, where implementation takes place in a variety of settings and targets the general public, creating partnerships with external organizations are often a key to success [32]. Thus, program administrators should facilitate collaborations where possible and emphasize the importance of community networks in program training. Researchers using CFIR to evaluate community-based programs should explore possible external influences on implementation.

Other personal attributes and executing were distinguishing constructs in our study and Kegler and colleagues found them to be salient [30], but others have not had the same result [18, 21, 26, 29]. Practically, personality attributes, like tolerance for stress and resourcefulness, cannot be controlled or translated to program design to improve implementation. Still, these attributes should be considered when developing training materials. The executing construct matched implementation scores in that low implementation programs had more negative ratings for executing and high implementation programs had positive ratings. While this coding method created overlap in the data, it captured implementation details not used in the implementation scoring. As suggested by Damschroder, we found this construct to be more descriptive than explanatory [12].

### Strengths and limitations

This study design has several strengths. First, it used an integrated mixed methods design to evaluate program implementation and outcomes, with each method enhancing the other. For example, the degree of implementation score (quantitative) was a better measure because the qualitative data helped to determine if each program component was fully implemented. Further, creating “hybrid” [15] research designs that assess both implementation and outcomes accelerates research translation, improves public health impact, and ensures accurate interpretation of program outcomes [14, 15, 17]. Using the CFIR throughout the research process from developing the research methodology and interview guide to analyzing the data is another notable strength [20]. It is a comprehensive framework [12], making it easier for conclusions to be drawn across studies regarding which constructs may be the most important for implementation and under what circumstances, a key question for community health educators. A final strength of the study is that all 13 eligible C4L instructors implementing the program were interviewed.

While the study has several strengths, some limitations should be noted. One limitation was the use of instructor self-report for implementation scoring measures rather than direct observation, which could have introduced social desirability bias [33]. Program observation was not feasible with the resources available for this study but using the interview narrative helped the research team mitigate this bias. Another limitation was interviewing only Extension professionals. Interviewing ACS navigators (formally appointed external implementation leaders) and individuals from partnering organizations (external change agents) would have provided more implementation perspectives. Future studies should seek other stakeholders to provide a comprehensive picture of implementation. Finally, this study may not be widely generalizable, since it details a unique community-based cancer prevention program. Still, our results contribute to the growing implementation science literature, including the CFIR literature, and may provide considerations for community health promotion program administrators that would enhance implementation and contribute to greater public health impact.

## Conclusions

This study provides insight into the implementation of a community-based health promotion program, how implementation is related to participant outcomes, and outlines barriers and facilitators to implementation that can be addressed to improve outcomes. This evaluation revealed variation in the degree of implementation of a long-standing program in which all instructors are provided the same training and resources. Therefore, it is important to monitor implementation of ongoing programs, as it informs efforts needed for continued effectiveness and identifies sources of program drift. Analyses showed that implementation and insurance status were significantly associated with improved intention to change some cancer preventive behaviors. Exploring program fidelity and participant characteristics can help program administrators understand for whom and under what conditions an intervention may be successful in improving behaviors. Other intervention characteristics should also be considered to improve implementation and participant outcomes, including program training, the availability of training material, interventionist experience, and program material quality and design. Ensuring a program is compatible with workflow and organizational values is also beneficial. In addition, programs that incorporate external organizations should foster supportive communication and collaboration.

Lastly, this study adds to the CFIR literature by using it to evaluate a well-established community intervention. The CFIR was found to be appropriate for the themes found in the qualitative data analysis. However, using CFIR for this type of intervention revealed distinguishing constructs that were different than those found in studies of newly-implemented, clinical interventions, and those interventions being translated from clinical trials to community contexts. Our analysis revealed distinguishing constructs that highlighted the role of program experience and relationships with external organizations which were different from those found in studies of newly-implemented and/or clinical interventions. As the CFIR is intended to be used across multiple contexts, future research using the CFIR is needed to determine if these constructs are context-specific or relevant only to the C4L program, and thus, if the use of the CFIR should be limited to programs that are being newly implemented, implemented in clinical settings, or were developed in the clinic and are being translated to communities.

## Additional files

Additional file 1: Cooking for a Lifetime of Cancer Prevention (C4L) Program Instructor Training Agenda. Agenda for training of program instructors. (PDF 164 kb)

Additional file 2: Cooking for a Lifetime of Cancer Prevention (C4L) Instructor Interview Guide. Interview guide for semi-structured interviews of C4L program instructors. (PDF 136 kb)

## Data Availability

The datasets supporting conclusions of this article are not available publicly due to containing sensitive information, but are available from the corresponding author on reasonable request.

## Abbreviations

ACS: American Cancer Society
C4L: Cooking for a Lifetime Cancer Prevention Cooking School
CES: Cooperative Extension System
CFIR: Consolidated Framework for Implementation Research
FACS: Family and Consumer Sciences
FIT: Fecal Immunochemical Test
FOBT: Fecal Occult Blood Test
GBCCP: Georgia Breast and Cervical Cancer Program
VA: Veterans Affairs
