# Supplementary material for: Mixed methods evaluation of implementation and outcomes in a community-based cancer prevention intervention
Source: BMC Public Health. 2019 Aug 5;19:1051. doi: 10.1186/s12889-019-7315-y (PMC6683347; doi:10.1186/s12889-019-7315-y)
Supplement: Supplementary file 1 — Cooking for a Lifetime of Cancer Prevention (C4L) Program Instructor Training Agenda. Agenda for training of program instructors. (PDF 164 kb) [file 12889_2019_7315_MOESM1_ESM.pdf]

Additional File 1. Cooking for a Lifetime of Cancer Prevention (C4L) Program Instructor Training Agenda

Training for Cooking for a Lifetime of Cancer Prevention (C4L)

Wednesday, November 9, 2016
Macon, GA

9:30 a.m. – 3:30 p.m.

9:30 a.m. – 9:45 a.m.	Welcome/Introduction
9:45 a.m. – 10:00 a.m.	UGA & ACS/NBCCEDP Partnership
10:00 a.m. – 10:30 a.m.	Overview of C4L Format/Delivery
10:30 a.m. – 11:00 a.m.	ACS Cancer Presentation (reminder of ACS Guidelines/BCCP requirements)
11:00 a.m. – 12:00 p.m.	UGA Cancer Prevention Presentation
12:00 p.m. – 12:45 p.m.	Working Lunch
12:45 p.m. – 1:15 p.m.	C4L Evaluation – changes, consent forms, incentives
1:15 p.m. – 1:45 p.m.	Recruiting the target audience: women without insurance
1:45 p.m. – 2:30 p.m.	Planning your programs
2:30 p.m. – 3:00 p.m.	Sharing Your Plans
3:00 p.m. – 3:30 p.m.	Q & A/Wrap up
