# Supplementary material for: Mixed methods evaluation of implementation and outcomes in a community-based cancer prevention intervention
Source: BMC Public Health. 2019 Aug 5;19:1051. doi: 10.1186/s12889-019-7315-y (PMC6683347; doi:10.1186/s12889-019-7315-y)
Supplement: Supplementary file 2 — Cooking for a Lifetime of Cancer Prevention (C4L) Instructor Interview Guide. Interview guide for semi-structured interviews of C4L program instructors. (PDF 136 kb) [file 12889_2019_7315_MOESM2_ESM.pdf]

Additional file 2. Cooking for a Lifetime of Cancer Prevention (C4L) Instructor Interview Guide

	Interview Questions	Goal of questions
	**I will begin by asking you a few introductory questions about yourself and the inclusion of Cancer Cooking School into your programming.**	
Work Experience	**How many years have you worked for Cooperative Extension?**<br>• How long have you been working in your current county?<br>• How many years have you worked as a FACS Extension Agent?<br><br>**Was this your first Cancer Cooking School*? YES / NO**<br>• [If No] How many years have you participated in the program in the past?<br><br>**How many schools have you done this year?**<br>• [If more than one] Great. For the purposes of this interview, I would like to focus on the first Cancer Cooking School you did this year.	Characteristics of individuals<br><br>*To learn the interviewee's background to understand their experience with Extension and C4L program.*
Program Need	**Is there a strong need for the Cancer Cooking School in your community?**<br>• Is there a reason why you feel this way?<br><br>**How does the Cancer Cooking School fit with your goals and mission as a FACS agent?**	Inner Setting<br><br>*To learn if the agent is motivated by a perceived need for the CCS. (implementation climate, tension for change)*
	**Does the Cancer Cooking School compliment your current programming?**<br>• In what ways?	*To learn if C4L makes sense with other Extension programming. (compatibility)*

	**Why did you pick Cancer Cooking School over other opportunities?**<br>• Did you decide not to do another program so you could do Cancer Cooking School?	*To learn what influences the agent to commit to C4L, and if it must replace other programs.*
	**Finally, on a scale of 1-5, with 1 being "implementation was poor" to 5 being "implementation was excellent," how well do you think the Cancer Cooking School was implemented?**<br>What led you to choose this number instead of a (higher, lower) number?	
	**I would now like to discuss your process for planning the school, including recruiting the target audience. I would like to know what methods you employed and if they seemed to be helpful.**	
Planning	**Can you describe your plan for implementing the Cancer Cooking School?**<br>• How detailed was the plan?<br><br>**Was anyone else involved in the planning process?**<br>• What was his/her/their role?<br>• How engaged were these individuals?<br>• How often did you communicate with these partners?<br><br>**What role did the plan of work you completed at training play in your planning of the Cancer Cooking School?**<br><br>**Were there revisions or refinements to the plan?**	<u>Process</u><br><br>*To learn if the agent made any additional plans, timelines, or checklists to aid in planning the school. (planning)*<br><br>*To understand the partnership between the Extension agent and the ACS or Health Dept. representative.*<br><br>*To understand if the plan of work activity during training is helpful and serves to shape the actual C4L.*

Recruitment	**How did you recruit and communicate with potential participants of the Cancer Cooking School? Describe this process.**<br>• What methods were used?<br>• How often / how long were advertisements run? (i.e. if newspaper advertisement, when was it run, how often, proximity to CCS location)<br>• Who was involved?<br>• Was the screening tool used? (how, by whom)<br><br>**Did you feel that the recruitment process was successful?**<br>• [If Yes] What made it successful?<br>• [If No] How could it be improved?	Process<br><br>*To understand all of the methods used to reach the target audience. (intervention participants)*
Implementation	**Now we are going to talk about your experience on the day of the Cancer Cooking School program. Many of these will be simple Yes/No questions. Remember, you will not be in trouble for any of the responses that you give. We want to know about your honest experience. First,**<br><br>**Have you sent in your evaluations?**<br>• [If Yes] Did you include the coversheet?	
	**YES – Great, thank you for doing that! We will talk through some of this information again briefly, because I have a few additional questions related to the program.**<br><br>**NO – That is fine. Would you mind if we talk through the questions asked on the coversheet during the interview?**	

Where did you conduct your school? (i.e., the event location)

How many people registered?

How many people attended?

Was an ACS Navigator Present?

- [If Yes] Who is your client navigator?
- [If No] Was someone else available?
    - [If Yes] Who was available?

Was the ACS presentation given?

- [If No] Why?

Was the Extension presentation given?

- [If No] Why?

Were the consent forms completed?

- [If Yes] How many?

Was the consent information read to participants or did they read it themselves?

Did you have difficulty collecting informed consent documents?

- [If Yes] Why do you think it was difficult?
- [If No] What do you think made the process successful?

Were evaluations completed?

- [If Yes] How many?

<u>Process</u>

To understand how the activities that are central to C4L were executed, and if any components presented problems.

(executing)

	**Were recipes demonstrated?**<br>• How many?<br>• Which ones?<br>• When during the cooking school did you demonstrate the recipes?<br>• Is there anything else you want to tell me about the recipe demonstration and tasting?<br><br>**Were gift cards or door prizes drawn for?**<br>• [If Yes] What were the prizes?<br>• When was the drawing done?<br>• [If No] Why?<br><br>**What issues or complications arose during the Cancer Cooking School?**<br>• How did you handle this?<br><br>**Did you have to make changes or alterations to the CCS in order for it to work effectively?**<br>• [If Yes] What changes did you make?<br>• Were they effective?<br>• Why did you make those changes?<br><br>**Were you able to implement the Cancer Cooking School the way you had planned?**<br>• Why or why not?	
	**Thank you for providing that insight into how the Cooking School went for you. In the next few questions, I would like to discuss the current resources available for the program, like the training session, ACS grant money, and print materials. I would also like to know your overall thoughts about the Cancer Cooking School program.**	

Preparedness / Resources	**Did you feel prepared to implement the Cancer Cooking School?**<br>• [If Yes] What made you feel prepared?<br>• [If No] What would have made you feel more prepared?	<u>Characteristics of individuals</u><br><br>*To learn if the agent training was adequate, if agents still felt uneasy about certain components. (individual stages of change)*
	**How did you feel about implementing the Cancer Cooking School?**<br>• Did you have feelings of anticipation? Stress? Enthusiasm?<br>• Why?<br><br>**On a scale of 1 to 5, with 1 being “not at all confident” and 5 being “extremely confident,” how confident were you that you would be able to successfully implement the CCS?**<br>• What would make you feel more confident? (e.g., 4 instead of a 2)<br><br>**Did you feel that the materials provided for the program (PowerPoint, handouts, cookbook) were adequate?**<br>• Did you find that some materials were not needed?<br>• What other materials would be helpful?<br>• Do you have suggestions for how the materials could be improved?<br><br>**Did you have sufficient resources to implement and administer the Cancer Cooking School?**<br>• [If Yes] Are there any other resources you would like to receive?<br>• [If No] What other resources you would like to receive?	*To understand how agents view C4L, is it enjoyable for them or stressful. (knowledge and beliefs about intervention)*<br><br>*(self-efficacy)*<br><br><u>Intervention characteristics</u><br><br>*To learn if all of the materials are useful / necessary. Do agents use everything we provide? (design quality and packaging)*<br><br>*Resources could include training, time, money, or materials to use in the intervention. (cost)*

	**Did you have grant money left over?**<br><br>**Overall, did it take you more, less, or about the same amount of time you expected to implement the Cancer Cooking School?**	
Overall	**Was the number of schools you applied for an appropriate goal for you?**<br>• Why or why not?<br><br>**Did you feel that the participants enjoyed the Cancer Cooking School?**<br>• What part of the class seemed to be their favorite?<br>• Were there parts of the class that they did not seem to enjoy?<br>• Is there anything that you feel would make the class more enjoyable?	*(complexity)*<br><br>Process<br><br>*To learn about the agent's perception of the audience's reaction to the program. Were certain components received negatively / positively? (intervention participants)*
	**Did you think the Cancer Cooking School was effective?**<br>• Why or why not?<br><br>**On a scale of 1 to 5, with 1 being "not at all successful" and 5 being "extremely successful", how successful do you believe the Cancer Cooking School was?**<br>• What do you think would have made the school more successful? (e.g., 4 instead of a 2)<br><br>**If you were to do a Cancer Cooking School next year, would you do anything differently?**<br><br>**Overall, how well do you think the Cancer Cooking School fits with the goals of FACS Extension Nutrition and Chronic Disease Prevention / Health?**	Characteristics of Individuals *(knowledge and beliefs about intervention)*<br><br>Process *(executing)*<br><br>Inner Setting *(compatibility)*

*Internally, the program is referred to as "Cancer Cooking School," and thus, that terminology was used with the Extension Professionals (C4L instructors) during the interviews.
